# Phylogeography of *Toona ciliata* (Meliaceae) Complex in China Inferred from Cytonuclear Markers

**DOI:** 10.3390/genes14010116

**Published:** 2022-12-31

**Authors:** Yu Xiao, Xin-Xin Zhang, Ying Hu, Xi Wang, Pei Li, Zi-Han He, Yan-Wen Lv, Xiao-Yang Chen, Xin-Sheng Hu

**Affiliations:** 1 College of Forestry and Landscape Architecture, South China Agricultural University, Guangzhou 510642, China; 2 Guangdong Key Laboratory for Innovative Development and Utilization of Forest Plant Germplasm, South China Agricultural University, Guangzhou 510642, China

**Keywords:** *Toona ciliata*, population structure, gene flow, isolation by distance, genetic conservation

## Abstract

*Toona ciliata* is an important timber species but is recognized as an endangered species at level II in China. Its genetic conservation is of increasing concern. Provenance trials and other breeding programs were conducted to develop seed transfer rules and multiplications. Here, we investigated twenty-nine populations sampled across the natural distribution of the *T. ciliata* complex using mtDNA and nrDNA ITS (ribosomal internal transcribed spacer) markers. Haplotype diversity was *h* = 0.190 ± 0.202 and nucleotide diversity was *π* = 0.000383 ± 0.000536 for mtDNA marker. Nucleotide diversity for ITS sequences was 0.00837 ± 0.000783. Haplotypes exhibited phylogeographic structure in spatial distribution. The extent of genetic differentiation was significant (*F_st_* = 0.6994 ± 0.0079 for ITS and 0.8870 ± 0.0077 for mtDNA marker). Isolation by distance (IBD) and by elevation (IBE) occurred among populations. Phylogenetic relationships from mtDNA marker indicated three genetically distinct regions, each without IBD effects. Compared with pollen flow, seed flow was strongly impeded in the western region, but extensive in the central region, and less impeded in the eastern region. Most populations did not exhibit expansion, with only a few populations showing expansion after bottleneck effects. We discussed a strategy of region-based genetic conservation and proposed to conserve multiple populations in the western and eastern regions and a few populations in the central region.

## 1. Introduction

*T. ciliata* is a deciduous or semi-deciduous tree in the *Toona* genus of the Meliaceae family and an important timber species in southern China [1]. It is naturally distributed in subtropical and tropical regions, including India, Malaysia, Indonesia, and other tropical regions. The species mainly occurs at elevations between 300 and 2600 m in China and on some arid and barren hillsides, with relatively wet and fertile soils and an annual average temperature of 15~22 °C. The species can attain 35 m in height and about 1 m in diameter at breast height (DBH) under a habitat of wet acid soil or calcareous soil with good drainage. One remarkable characteristic is that *T. ciliata* has high-quality wood, with a tough and straight texture, beautiful patterns, dark reddish-brown heartwood, and light sapwood, and is also known as Chinese mahogany [2,3,4]. *T. ciliata* is an ideal material for art processes and antique furniture in China and has a significant economic value [5]. However, *T. ciliata* is currently recognized as an endangered species at level II because of over exploitation and low natural regeneration [6,7]. Its genetic conservation is now of increasing concern and has been the subject of provenance trials aimed at delineating seed zones and developing seed transfer rules [8]. Other breeding programs have also been extensively conducted to improve multiplications of this species [9,10].

Based on the leaf and flower morphological traits, *T. ciliata* is taxonomically classified into five varieties, namely *T. ciliata* var. *ciliata*, *T. ciliata* var. *yunnanensis*, *T. ciliata* var. *pubescens*, *T. ciliata* var. *sublaxiflora*, and *T. ciliata* var. *henryi* [1]. Although there is debate about the taxonomic status of these five taxa, classification of these varieties could conceptually and practically complicate conservation of this endangered species [11]. Here, we bring these varieties together as the *T. ciliata* complex. Previous studies of population genetic structure were mostly conducted in terms of the *T. ciliata* complex rather than a single variety, showing significant population differentiation using either the sequence-related amplified polymorphism (SRAP) ($F_{st}$ = 0.7924) [12] or the simple sequence repeat (SSR) markers ($F_{st}$ = 0.35) [13]. There were significant effects of isolation by distance (IBD) across range-wide populations of *T. ciliata* complex. Zhan et al. further classified the range-wide populations into western and eastern regions across the natural distribution of *T. ciliata* complex [13]. *T. ciliata* var. *pubescens* was probably the only variety whose population genetic structure was studied using molecular markers [14]. Nevertheless, the ecological and evolutionary processes underlying the pattern of population genetic diversity of *T. ciliata* complex have not been studied in depth, which limits us to develop an appropriate strategy for genetic conservation.

Studies pertinent to genetic conservation of *T. ciliata* complex also include its reproductive system which was shown to be a predominantly outcrossing system, with selfing and inbreeding [15]. Inbreeding depression occurred in natural populations and could result in a decline in population density of natural forests and low regeneration [7]. In addition, previous phylogeny studies indicated that *T. ciliata* was divergent from other species of the same family at a wide range of evolutionary times (7.4–48.4 Mya) [16,17,18,19]. We recently showed that *T. ciliata* was divergent from its close species *T. sinensis* of the same genus at about 6–25 Mya using the whole genome sequences [20,21]. These studies help us to understand the taxonomic position of *T. ciliata* in the Meliaceae family and the significance of conserving *T. ciliata* from a broader perspective.

The purposes of this study were to investigate phylogeographic variation across range-wide populations of *T. ciliata* complex, with emphasis on inferring gene flow underlying the observed phylogenetic pattern, and to discuss genetic conservation of the species. Previous studies with SRAP and SSR markers were mainly confined to population structure of *T. ciliata* complex and did not infer the evolutionary processes underlying the observed pattern [12,13]. This provided us with preliminary information on making a strategy of genetic conservation but limited our insights into the evolutionary processes. In this study, we investigated the population genetic structure of *T. ciliata* complex using both nuclear and organelle genetic markers. For nuclear genomes, we selected a ribosomal internal transcribed spacer (ITS) marker that has been applied to population genetic analysis of plant species [22,23] and phylogeny among species in Meliaceae [16]. Its overall variability is generally lower than that of SSR markers but could likely be greater than that of SRAP markers [24]. For organelle genomes, we used both chloroplast (cpDNA) and mitochondrial DNA (mtDNA) markers that are maternally inherited. Here, we reported the results derived from both nrDNA and mtDNA markers. The results from cpDNA markers will be present separately. Gene flow is mediated through different vectors for nuclear ITS markers (both seed and pollen flow) versus for mtDNA markers (only seed flow) among populations in the *T. ciliata* complex. A combination of cytonuclear markers helps to infer the relative rate of pollen to seed flow among natural populations [25,26].

In addition, we investigated the potential historical range expansion or contraction, which could provide additional information on genetic conservation. Although postglacial impacts on the distribution of *T. ciliata* complex are unknown in South China, potential historical changes in populations size could yield different extents of genetic variation, as implied by postglacial colonization of some plant species [27,28,29,30]. The bottleneck effect, among common phenomena in species’ range shifting, can produce several population genetic patterns, such as a reduction in haplotype and nucleotide diversity [24]. Through analyzing these patterns, we tested the bottleneck effects of all investigated populations of the *T. ciliata* complex. This information was then combined with population structure and gene flow to develop a strategy for genetic conservation.

## 2. Materials and Methods

### 2.1. Population Sampling and DNA Extraction

**Figure 1:**
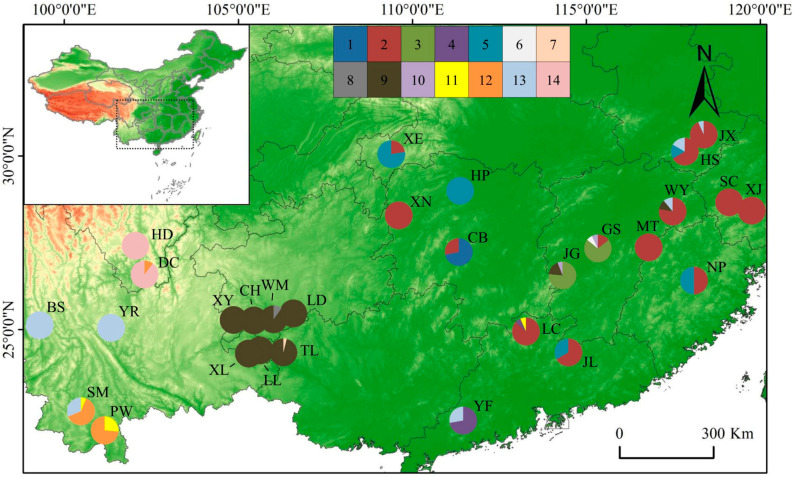
A map shows the twenty-nine sample sites and the geographic distribution of the mtDNA haplotypes that were derived from concatenated sequences of *cox*1−*nad*1 and *26S*−*rRNA*−*tRNA*− Leu. Pie charts show different proportions of haplotypes within each of 29 populations of *T. ciliata* complex. H*i* (*i* = 1,2, …, 14) represents the *i*th haplotype. Each of fourteen colors in pie charts represents one haplotype (H1–H14).

Leaf samples were collected from 29 populations in 11 provinces, covering the range-wide distribution of *T. ciliata* complex in China (Table 1, Figure 1). Parent trees for collecting leaf samples were separated at least 50 m away in natural forest stands. A total of 500 individuals were analyzed in this study, ranging from 3 to 30 samples per population. The sample size in this study was less than that used by Li et al. [12] where a total of 853 individuals were analyzed. Most DNA samples extracted from healthy leaves were prepared and frozen by Li et al. [12]. Supplementary samples were collected in 2018 from the provenance trials at Zengcheng Experiment Station (113°37′ E, 23°14′ N, and 20.3 m above sea level) and Yuejin North Experiment Station, South China Agricultural University (113°37′ E, 23°16′ N, and 42.3 m above sea level). Supplementary DNA samples were extracted from leaves on living plants by following the CTAB 2X protocol [31]. The quality of DNA extraction was checked by 0.8% (*w*/*v*) agarose gel electrophoresis. All quantified DNA samples were stored at −20 °C for polymerase chain reaction (PCR) amplification.

### 2.2. Primer Screening, Amplification and Sequencing

For the mitochondrial genome, we tested twenty pairs of primers, with fourteen pairs from the literature [32,33,34] and six pairs designed in this study. Primer sequences were detailed in Table S1.

For the nuclear genome, we selected ITS fragment as marker [35], which covers the region of ITS5a-ITS4. The forward and reverse primers are CCTTATCATTTAGAGGAAGGAG and TCCTCCGCTTATTGATATGC, respectively.

The PCR amplification was carried out in a 25 μL reaction volume that contained 1μL template DNA, 1 μL of each forward and reverse primer, 12.5 μL 2×ES Taq Mastermix (including Es Taq DNA polymerase, 3 mM MgCl_2_ and 400 μM each dNTP) and 9.5 μL ddH_2_O. The amplifications were performed in Dongsheng Thermal Cycler (EDC-810, Suzhou, China). The PCR amplification for ITS and mtDNA primers was conducted as follows: preheating at 95 °C for 3 min, 32 cycles at 95 °C for 1 min, annealing at 50 °C for 1 min, and elongation at 72 °C for 1.5 min, followed by a final extension at 72 °C for 30 s. The amplified DNA fragments were subsequently sent to Sangon Biotech (Shanghai) for Sanger sequencing. All sequence data obtained by the company were checked with Chromatogram Explorer 3.2 software. The sequences with high quality and no mixed peak signals were used for downstream analyses.

### 2.3. Analysis of Genetic Diversity

The sequenced fragments of both ITS and mtDNA markers from 29 populations were aligned by MEGA7 [36], removing the parts with heterozygous interferences caused by unstable signals at the front and end. For indel (gap) polymorphisms observed in mtDNA fragments, the insertion and deletion were treated as the fifth nucleotide site and coded as mutant substitutions. Ultimately, we obtained two datasets from 29 populations for analysis, one for concatenated sequences from mitochondrial genome and the other from nuclear ITS sequences.

We used DNAsp v5 [37] to estimate haplotype diversity (*h*), nucleotide diversity (π), and effective population size-scaled mutation ($\theta =4N_{e}\mu$) [38,39,40]. TCS v1.21 [41] was used to draw the evolutionary network among mitochondrial haplotypes.

### 2.4. Population Genetic Structure

Population genetic differentiation was measured using several indices, including $F_{st}$ [42], $N_{st}$ [43,44], $G_{st}$ [40] and $\phi_{st}$ [45]. Both $G_{st}$ and $\phi_{st}$ are analogous to $F_{st}$ in biological meaning except using for multiple alleles (>2) and haploid sequence data, respectively. We used Arlequin v3.0 [46] and DNAsp v5 [37] to estimate these parameters. We tested whether $N_{st}$ was larger than $G_{st}$ or not to infer if a phylogeographic structure occurred [43].

Isolation by distance (IBD) was tested through regression analysis of $F_{st}/\left(1-F_{st}\right)$ on the logarithm of geographic distance [47,48]:

(1) $$\frac{F_{st}}{1-F_{st}}=a+b\cdot \ln\left(\mathrm{geographic}\;\mathrm{distance}\right)$$

A significant difference of the regression coefficient *b* from zero indicates the presence of IBD effects. Note that the geographical distance between two populations was calculated using their longitude and latitude coordinates (Table 1). A Mantel’s test was also conducted to examine the relationship between $F_{st}$ and the geographic distance [49].

Correlation between $F_{st}$ and the difference in elevation between populations was tested to check if there were effects of isolation by elevation (IBE).

Nei’s genetic distance was calculated between populations [50]. Phylogenetic relationships among individuals and among populations were reconstructed by MEGA 7 [36].

### 2.5. Population Demography

To identify demographic changes, we tested neutrality of markers using Tajima’s *D* [51] and Fu’s *F* [52] tests. Analysis of mismatch distribution was performed by Arlequin v3.0 [46]. Under the null hypothesis that a population expanded after bottleneck effects, significant negative values of Tajima’s *D* and Fu’s *F* statistics could be expected.

We analyzed mismatch distribution using the ITS sequences for those populations with significant Tajima’s *D* or Fu’s *F* values [46]. A unimodal distribution of the frequency of observed number of pairwise different sites, which fits for a single-peaked Poisson distribution, would signal population expansion after bottleneck effects. Statistical testing was ducted using the sum of square deviations (SSD) and Harpending’s raggedness index (Rag) to check if the expected SSD (or Rag) was greater than the observed SSD (or Rag) [53]. Other parameters were estimated, including $\theta_{0}$ = 2$N_{0}\mu$, $\theta_{1}$ = 2$N_{1}\mu$, where $N_{0}$ and $N_{1}$ are the population sizes before and after population expansion, and τ (t) , the time elapsed since a sudden expansion.

### 2.6. Ratio of Pollen to Seed Flow

We assessed the extent of gene flow of *T. ciliata* complex based on population genetic structure. Under the assumption of the classical island model [54], $F_{st}$ for haploid and diploid markers is expressed as *F_st_*_(haploid)_ = 1/(1 + 2*N*_e_*m*) and *F_st_*_(diploid)_ = 1/(1 + 4*N*_e_*m*), respectively, where *m* is the migration rate and *N_e_* is the effective population size. MtDNA marker is maternally inherited and only seed flow contributes to its gene flow. The nrDNA ITS marker is biparentally inherited and both seed and pollen flow contribute to its gene flow. According to Ennos [25] and Hu and Ennos [26], population genetic differentiation coefficients for nuclear markers, denoted by$\;F_{st\left(n\right)}$, and for maternal markers, denoted by $F_{st\left(m\right)}$, are respectively given by

(2) $$F_{st\left(n\right)}=\frac{1}{1+4N_{e}\left(m_{s}+m_{p}/2\right)}$$

(3) $$F_{st\left(m\right)}=\frac{1}{1+2N_{e}m_{s}}$$

where *m_s_* is the rate of seed flow and *m_p_* is the rate of pollen flow. From Ennos [25], the relative rate of pollen to seed flow is estimated from Equations (2) and (3):

(4) $$\frac{m_{p}}{m_{s}}=\frac{\left(1-F_{st\left(n\right)}\right)F_{st\left(m\right)}}{\left(1-F_{st\left(m\right)}\right)F_{st\left(n\right)}}-2.$$

To estimate the standard deviation of the point estimate of *m_p_/m_s_*, let $X=\left(1-F_{st\left(n\right)}\right)F_{st\left(m\right)}$ and $Y=\left(1-F_{st\left(m\right)}\right)F_{st\left(n\right)}$. We assume that the covariance cov($F_{st\left(n\right)}$, $F_{st\left(m\right)}$) is negligible, similar to Hu et al. [55]. Therefore, the variances of X and Y are derived as

(5) $$V\left(X\right)={\bar{F}}_{st\left(m\right)}^{2}V\left(F_{st\left(n\right)}\right)+{\left(1-{\bar{F}}_{st\left(n\right)}\right)}^{2}V\left(F_{st\left(m\right)}\right)+V\left(F_{st\left(m\right)}\right)V\left(F_{st\left(n\right)}\right),$$

(6) $$V\left(Y\right)={\bar{F}}_{st\left(n\right)}^{2}V\left(F_{st\left(m\right)}\right)+{\left(1-{\bar{F}}_{st\left(m\right)}\right)}^{2}V\left(F_{st\left(n\right)}\right)+V\left(F_{st\left(m\right)}\right)V\left(F_{st\left(n\right)}\right).$$

The variance $V\left(F_{st\left(m\right)}F_{st\left(n\right)}\right)$ is

(7) $$V\left(F_{st\left(m\right)}F_{st\left(n\right)}\right)={\bar{F}}_{st\left(m\right)}^{2}V\left(F_{st\left(n\right)}\right)+{\bar{F}}_{st\left(n\right)}^{2}V\left(F_{st\left(m\right)}\right)+V\left(F_{st\left(m\right)}\right)V\left(F_{st\left(n\right)}\right).$$

The covariance cov(X,Y) is estimated as

(8) $$cov\left(X,\;Y\right)=-{\bar{F}}_{st}{}_{n}\left(1-{\bar{F}}_{st\left(m\right)}\right)V\left(F_{st\left(m\right)}\right)-{\bar{F}}_{st\left(m\right)}\left(1-{\bar{F}}_{st}{}_{n}\right)V\left(F_{st\left(n\right)}\right)\;+V\left(F_{st\left(m\right)}\right)V\left(F_{st\left(n\right)}\right)$$

From Lynch and Walsh [56], the variance of *m_p_/m_s_* is estimated by

(9) $$V\left(\frac{m_{p}}{m_{s}}\right)=V\left(\frac{X}{Y}\right)={\left(\frac{\bar{X}}{\bar{Y}}\right)}^{2}\left[\frac{V\left(X\right)}{{\bar{X}}^{2}}+\frac{V\left(Y\right)}{{\bar{Y}}^{2}}-\frac{2cov\left(X,\;Y\right)}{\bar{X}\bar{Y}}\right].$$

The Jacknife method was applied to estimate variances of $F_{st\left(m\right)}$ and $F_{st\left(n\right)}$ with FSTAT [57], which were then used to calculate the variance $V\;\left(m_{P}/m_{S}\right)$ according to Equation (9).

## 3. Results

### 3.1. Haplotype Analysis

**Figure 2:**
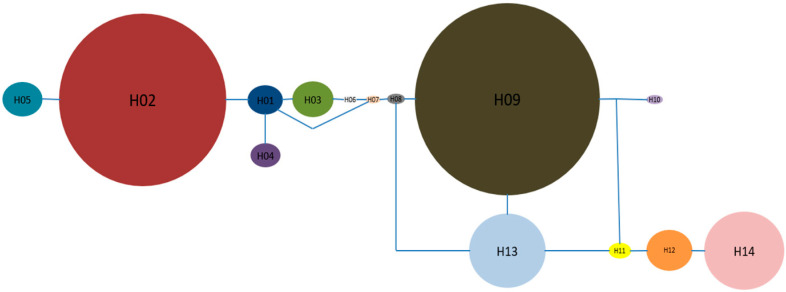
A network of mitochondrial haplotypes that were identified from concatenated sequences of *cox1−nad*1 and *26S−rRNA−tRNA−*Leu. The circle sizes are proportional to the haplotype frequencies in 500 individuals. Code H*i* (*i* = 1, 2, …,14) represents the *i*th haplotype and each haplotype is an unique combination of multiple alleles at polymorphic nucleotide sites.

Among the twenty pairs of mtDNA primers investigated (Table S1), two primers for *cox1−nad*1 and *26S−rRNA−tRNA−*Leu were successfully screened for generating polymorphisms among individuals. Sequences of these two fragments were aligned and concatenated to produce a length of 1242 bp for downstream analyses (Table S2 for 500 individuals). Across 500 sampled individuals, 14 haplotypes were identified (H1–H14) (Figure 2). The two most frequent haplotypes (H02 and H09) accounted for 55% of all samples. H2 was the most common haplotype and occurred in 12 populations. Figure 1 shows the geographical distribution of different haplotypes across 29 populations, with the number of haplotypes per population ranging from one to four (Figure 1).

Table 2 shows the haplotype and nucleotide diversity in 29 populations. The haplotype diversity ranged from 0.000 to 0.508, with the average diversity of 0.190 (±0.202). Population Simao (SM) in Yunnan Province, had the highest diversity, h = 0.5078, followed by Huangshan (HS, h = 0.50) in Anhui Province and Nanping (NP, h = 0.50) in Fujian Province. The nucleotide diversity was relatively higher in Yunfu (YF, π = 0.00171) in Guangdong Province, Jinggangshan (JG, 0.00173) in Jiangxi Province, and Wuyishan (WY, 0.00148) in Fujian Province, and less than 0.001 in the remaining twenty-nine populations. Average nucleotide diversity was 0.000383 (±0.000536).

We obtained 467 haploid ITS sequences after removing some sequences of low quality (Table S3). The length of aligned haploid ITS sequences was 622 bp. Nucleotide diversity ranged from 0.0011 in Xianju (XJ) in Zhejiang Province to 0.0322 in Baoshan (BS) in Yunnan Province, with the average diversity of 0.00837 (±0.000783). The effective population size-scaled mutation rate θ ranged from 0.0024 in Xianju (XJ) to 0.038 in BS, with the mean of 0.01262 (±0.009653). Populations HS (θ = 0.036) and WY (θ = 0.032) also maintained higher population diversity (Table 2).

### 3.2. Population Genetic Structure

Analysis of molecular variance (AMOVA) indicated that significant genetic differentiation occurred among populations (Table 3). Estimates of $\phi_{st}$ using mtDNA markers were 0.8858 for *cox1−nad*1, 0.8901 for *26S−rRNA−tRNA−*Leu, and 0.8884 for their concatenated sequences. The estimate of $\phi_{st}\;$using nrDNA ITS marker was 0.7143.

Population differentiation based on sequence divergence ($N_{st}$) for mtDNA marker was 0.8670, while population differentiation based on allele frequency ($G_{st}$) was 0.7744. $N_{st}$ was significantly greater than $G_{st}$ (*p* < 0.05). Likewise, the $N_{st}$ estimate for haploid nrDNA ITS sequences was 0.6957, which was significantly greater than $G_{st}$ (=0.0918). Both analyses indicated that phylogeographic structure occurred for haplotype distribution in space (Figure 1 for mtDNA marker).

**Figure 3:**
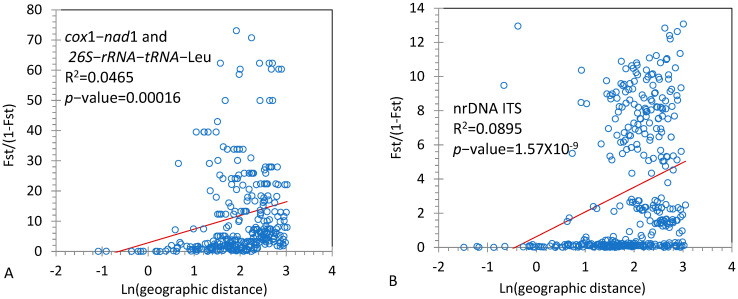
Effects of isolation by distance (IBD) among range-wide populations of *T. ciliata* complex. (**A**). Results from the concatenated sequences of *cox1−nad*1 and *26S*−*rRNA*−*tRNA*−Leu from mitochondrial genome; (**B**). Results from nrDNA ITS sequences.

Figure 3 indicates that a significant relationship existed between $F_{st}/\left(1-F_{st}\right)$ and geographic distance, where $F_{st}/\left(1-F_{st}\right)$ = 2.8412 + 4.5181 ln (distance), $R^{2}$ = 0.0464 (*p*-value = 0.00016) for the concatenated mtDNA sequences, and $F_{st}/\left(1-F_{st}\right)$ = 0.6275 + 1.4463 ln (distance), $R^{2}$ = 0.0895, and *p*-value = 1.57 × 10^−9^ for ITS sequences. These results indicated that significant IBD effects occurred among range-wide populations of *T. ciliata* complex.

**Figure 4:**
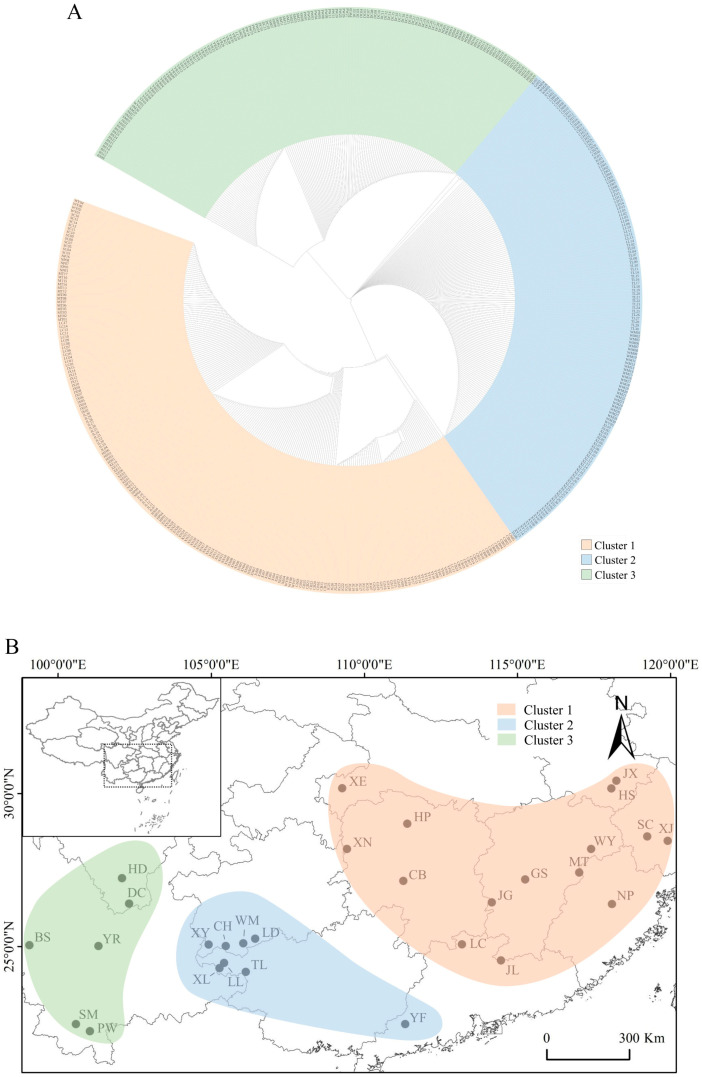
Phylogenetic relationships and geographic clusters of 29 populations. (**A**) is the phylogenetic relationships among 500 individuals derived from mtDNA markers, showing three clusters. (**B**) is the three regions of populations corresponding to the three clusters in the top figure.

Phylogenetic relationships among individuals using mtDNA markers indicated that three clusters were explicitly grouped among 500 individuals (Figure 4A), When mapped to their geographic positions, these three clusters respectively represented the eastern, central, and western regions of the natural distribution of *T. ciliata* complex (Figure 4B). For instance, the mtDNA haplotype H09 that was dominated in the central region did not occur in either the eastern region or the western region (Figure 1). The eastern region covered Anhui, Fujian, Hubei, Hunan, Jiangxi, Zhejiang, and Eastern Guangdong Provinces. The central region covered Guangxi, Guizhou, and Southwest Guangdong Provinces. The western region mainly included Sichuan and Yunnan Provinces. The mtDNA haplotype and nucleotide diversity, on average, were greater in the eastern region (h = 0.2511 ± 0.2068, π = 0.00052 ± 0.00057) than in the western region (h = 0.1803 ± 0.2227, π = 0.00021 ± 0.00031) or in the central region (h = 0.0819 ± 0.1439, π = 0.00025 ± 0.00059). However, the nucleotide diversity for ITS sequences was π = 0.00842 ± 0.0087 and θ = 0.0124 ± 0.0112 in the eastern region, π = 0.0069 ± 0.0022 and θ = 0.012 ± 0.0038 in the central region, and π = 0.0102 ± 0.0108 and θ = 0.0140 ± 0.0120 in the western region.

Regression analyses indicated that IBD effects were insignificant within each of three regions. However, significant IBD effects occurred among populations within the combined eastern and central regions (*p*-value = 7.8 × 10^−5^) or within the combined western and central regions (*p*-value = 6.7 × 10^−7^). This supported that the natural distribution of *T. ciliata* complex could be divided into three genetically differential groups in terms of mtDNA marker.

Analysis of genetic differentiation indicated that there was significant difference between the eastern and central regions ($\phi_{st}$ = 0.7799 for the mtDNA marker and 0.8106 for ITS marker, *p*-value < 0.001), between the central and western regions for mtDNA marker ($\phi_{st}$ = 0.6252, *p*-value < 0.001) but not for ITS marker ($\phi_{st}$ = 0.004, *p*-value = 0.06), and between the eastern and western regions ($\phi_{st}$ = 0.7100 for mtDNA marker, 0.7938 for ITS marker, *p*-value < 0.001). Differentiation among three regions was significant ($\phi_{st}$ = 0.9015 for mtDNA marker, 0.7143 for ITS marker, *p*-value < 0.001).

**Figure 5:**
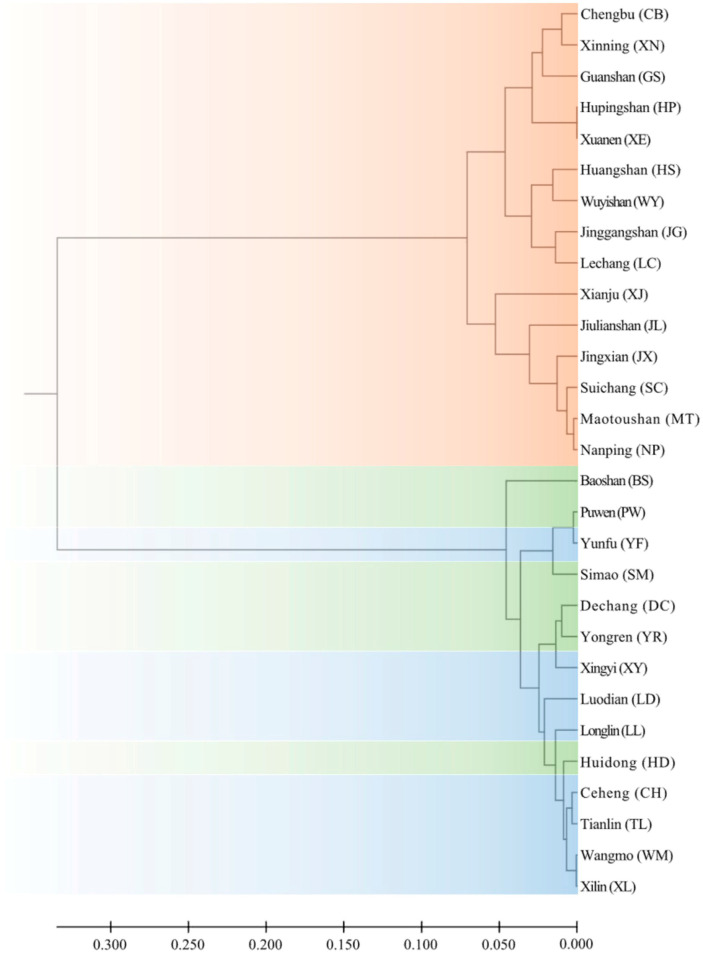
Phylogenetic relationships among 29 populations derived from nrDNA ITS sequences in *T. ciliata* complex. UPGMA (unweighted pair-group method using arithmetic average) was used to draw clusters. The populations in three colors correspond to the three clusters derived from the mtDNA marker in Figure 4.

Phylogenetic relationship from ITS sequences indicated that two clusters could be grouped among twenty-nine populations (Figure 5). One cluster corresponded to the eastern region derived from the concatenated mtDNA marker, while the other cluster corresponded to the combined central and western regions (Figure 4). Analysis of IBD effects indicated that there were no significant effects in the eastern region, $F_{st}/\left(1-F_{st}\right)$ = 0.1183 + 0.0246 ln (distance) ($R^{2}$ = 0.0096, *p*-value = 0.3189), but significant effects in the combined central and western region, $F_{st}/\left(1-F_{st}\right)$ = 0.0621 + 0.0356 ln (distance) ($R^{2}$ = 0.1211, *p*-value = 0.0007).

A significantly positive correlation occurred between $F_{st}$ and elevation with 29 populations (the correlation coefficient r = 0.2111, *p*-value = 0.00022 for the mtDNA marker; r = 0.1312, *p*-value = 0.0094 for the ITS marker). However, correlation between $F_{st}$ and elevation was insignificant in both the western (*r*=0.0176, *p*-value = 0.9566) and central (*r* = 0.2668, *p*-value = 0.4018) regions, but significant in the eastern region (*r* = −0.2644, *p*-value = 0.0219) for the mtDNA marker. Similarly, correlation between $F_{st}$ and elevation was insignificant in the western and central regions (*r* = 0.0198, *p*-value = 0.8554) but significant in eastern region (*r* = −0.2141, *p*-value = 0.0372) for the ITS marker.

### 3.3. Relative Rate of Pollen to Seed Flow

Based on population genetic differentiation derived from both ITS and mtDNA markers and Equations (4) and (9), we calculated the relative rate of pollen to seed flow (Table 4). Pollen flow was much greater than seed flow in the western region ($m_{P}/m_{S}=$ 31.4134 ± 13.2330) and in the combined western and central regions (47.9628 ± 0.8091). Both seed and pollen flow were comparable, or seed flow could be even more extensive than pollen flow in the central region (−1.495 ± 0.0454). Pollen flow was slightly greater than seed flow in the eastern region (3.2270 ± 2.5527) and in the global region (1.3741 ± 0.2925). In general, pollen flow is more frequent in the western region but less frequent in the eastern region in the natural distribution of *T. ciliata* complex.

### 3.4. Population Demographic Analyses

Neutrality test with the ITS sequences indicated that both Tajima’s *D* and Fu’s *F* values were negative in most populations (Table 2). Seven populations had significant Tajima’s *D* values (*p*-value < 0.05) while nine populations had significant Fu’s *F* values (*p*-value < −0.05). Only three populations exhibited significantly negative values of both Tajima’s *D* and Fu’s *F* values, including Xilin (XL) in Guangxi Province, Luodian (LD), and Wangmo (WM) in Guizhou Province. The three populations were in the central region and potentially underwent an expansion after bottleneck effects. In general, the whole population-based test did not show a significant departure from neutrality when the significance level was adjusted by Bonferroni correction ($\alpha^{\prime }$ = 0.05/29 = 0.0017).

**Figure 6:**
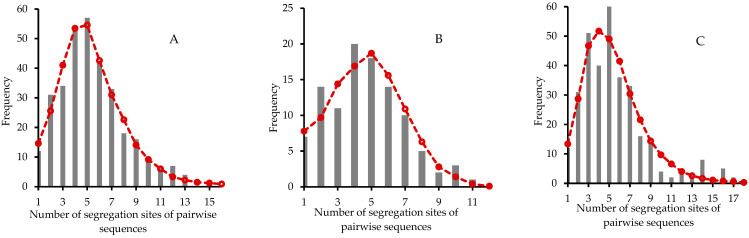
Analysis of mismatch distribution using haploid nrDNA ITS sequences. (**A**) population XL, (**B**) population LD, and (**C**) population WM. The bar lines are the observed frequencies under different numbers of segregation sites of pairwise sequences, whereas the red dashed lines represent the expected frequencies under a model of sudden population expansion [53].

Analysis of mismatch distribution indicated that populations with significant Tajima’s *D* or Fu’s *F* did not substantially expand (Table S2) although these populations except TL and XJ fit for a single-peaked Poisson distribution (Table S2; *p*-value > 0.05). Figure 6 shows the mismatch distribution of three populations (XL, LD and WM) with significant values of two tests. With population XL, the sum of squared deviation (SSD) was 0.002 (*p*-value = 0.84) and Harpending’s raggedness index (Rag) was 0.013 (*p*-value = 0.86). Other estimates of parameters were $\theta_{1}$ = 2$N_{1}\mu$ = 37.285 >$\;\theta_{0}$ = 2$N_{0}\mu$ = 1.552, and the time elapsed since a sudden expansion episode τ=2μt = 3.131. Similarly, with population LD, we obtained SSD = 0.003 (*p*-value = 0.94), Rag = 0.020 (*p*-value = 0.92), $\theta_{1}$ = 14.214, $\theta_{0}$ = 0.028, and τ  = 4.205. With population WM, estimates of demographic expansion were SSD = 0.005 (*p*-value = 0.53), Rag = 0.023 (*p*-value = 0.55), $\theta_{1}$ = 50.859, $\theta_{0}$ = 1.872, and τ  = 2.607. These local populations possibly underwent an expansion after bottleneck effects to different extents. Thus, in general, *T. ciliata* complex potentially underwent a weak expansion in some local regions but did not exhibit a substantially global expansion.

## 4. Discussion

### 4.1. Genetic Diversity

In this study, we used both nrDNA ITS and mtDNA markers to investigate phylogeographic pattern of *T. ciliata* complex. Both marker analyses indicated the presence of phylogeographic structure ($N_{st}>G_{st}$). Different haplotypes were not randomly distributed in space. The average haplotype diversity was h = 0.190 ± 0.202 and the average nucleotide diversity was π = 0.000383 ± 0.000536 for mtDNA markers. The average nucleotide diversity was π = 0.00837 ± 0.000783 for ITS sequences. The haplotype diversity for mtDNA marker was the highest in the eastern region, followed by the eastern region and then the central region. The genetic diversity in the central region was also the smallest among three regions for ITS sequences.

No significant deviation from neutrality was detected in nrDNA ITS sequences in most populations. The overall level of nucleotide diversity derived from ITS sequences in *T. ciliata* complex was greater than those found in other plant species, such as *Primula obconica* (average π = 0.00122 for Eastern group and 0.00739 for Yunnan group of Lineage A; 0.00144 for Sichuan group and 0.00164 for Central group of Lineage B) [58], *Tamarix Chinese* (π = 0.00217) [23] and *Achyranthes bidentata* (π = 0.00188) [59], but smaller than or comparable to *Spiraea alpina* (π ranging from 0 to 0.01) [22]. The overall level of haplotype or nucleotide diversity derived from mtDNA markers was relatively smaller than those found in other angiosperms, such as *Cucurbita moschata* (average π = 0.0023, h = 0.316) [60], *Medicago sativa* (h = 0.583 for whole cultivated pool and h = 0.821 for wild pool) [61], and *Fagus crenata* (h = 0.833) [62]. Our results suggest that a smaller genetic diversity of mtDNA marker but a certain level of genetic diversity of nrDNA ITS marker were present in the extant natural populations of this endangered species.

### 4.2. Population Genetic Structure

Analysis of genetic structure indicated that significant and substantial genetic variation occurred among populations within *T. ciliata* complex. The results from nrDNA ITS marker consolidate previous findings derived from nuclear SRAP and SSR markers [12,13] where two clusters of populations (eastern and western regions) were classified in the natural distribution of *T. ciliata* complex. However, the results from mtDNA markers suggest three genetically differential regions. These three regions have three levels of elevations that increase from the eastern region (617.34 ± 264.19 m) to the central region (732.37 ± 250.87 m) and to the western region (1407.67 ± 321.81 m). Although IBD effects were significant across 29 populations, they were absent within each of three regions in terms of mtDNA markers. This suggests that geographical distance did not significantly impede seed dispersal within each region, but globally impeded seed dispersal in the natural distribution of *T. ciliata* complex.

The difference in genetic structure between mtDNA and nrDNA markers is related to the effects of pollen flow on genetic variation of nrDNA markers and to the reproductive ecology of *T. ciliata*. Its predominantly outcrossing system enhances pollen flow [15]. Pollen dispersal is realized mainly through wind whereas seed dispersal is mediated by wind and animals in *T. ciliata*. Generally, pollen dispersal can travel a longer distance than seed dispersal for predominantly outcrossing species [25]. Gene flow for the maternally inherited mtDNA markers is mediated through seed dispersal only. As expected in theory [25,26], population genetic differentiation was greater for mtDNA markers than for nrDNA markers in western and eastern regions. However, this was not the case in the central region where $F_{st\left(n\right)}$ for the ITS marker was smaller than $F_{st\left(m\right)}$ for the mtDNA marker. One possible explanation is that both seed and pollen dispersal were extensive in the central region, yielding small population differentiation (Table 4). When seed flow was more restricted within the central region while pollen flow was less restricted, including alien genes immigrated from distant regions, this could lead $F_{st\left(n\right)}$ to be greater than $F_{st\left(m\right)}$. The populations investigated in the central region are located at elevations below 1200 m and are in the southern region between the Hengduan and Xuefeng Mountains. Physical barrier to seed dispersal could be weak in the south region. The insignificant correlations between $F_{st\left(n\right)}$ and elevation or between $F_{st\left(m\right)}$ and elevation support that elevation did not significantly affect seed or pollen flow in the central region. However, barrier would be strong to westward seed dispersal due to high plateaus and mountains at elevations above 1000 m. Thus, long distance gene flow from the central region to the western region is less frequent than the reverse directional gene flow. This could probably explain why $F_{st\left(n\right)}$ was greater than $F_{st\left(m\right)}$ in the central region.

In the western region, barriers to seed dispersal could be strong due to the Hengduan Mountains (7556 m above sea level at the highest peak). Populations investigated in this study cover both the north (HD, and DC) and south (SM and PW) regions of the Hengduan Mountains. Populations BS and YR are almost in the middle region of the Hengduan Mountains. Barrier to seed flow is expected to be greater than that to pollen flow although elevation did not significantly affect population genetic differentiation. This is because all populations except PW are located at comparable elevations (above 1300 m). As a result, the ratio of pollen to seed flow was much greater than 1 (Table 4 ).

In the eastern region, IBD effects were insignificant for both mtDNA and nrDNA ITS markers, implying that barriers to both seed and pollen flow were weak. Most investigated populations except WY are located at elevations below 1000 m. As expected, the ratio of pollen to seed flow was not much greater than 1, implying the presence of a certain level of seed flow among populations. The negative correlation between $F_{st\left(n\right)}$ and elevation, or between $F_{st\left(m\right)}$ and elevation, implies weak barriers to seed and pollen flow in the eastern region.

IBD effects were significant at the global level, implying limited seed and pollen flow. In addition, significant correlation between $F_{st\left(n\right)}$ (or $F_{st\left(m\right)}$) and elevation implies effects of elevation on population differentiation at the global level as well. This is analogous to the case of *Machilus pauhoi* [63], where high mountains as a physical barrier to gene flow generated substantial population genetic differentiation.

### 4.3. Genetic Conservation

Analyses of Tajima’s *D* and Li and Fu’s *F* imply that most populations did not undergo recent expansion. Only three populations (XL, LD, and WM) had significantly negative values of Tajima’s *D* and Fu and Li’s *F*. Analysis of mismatch distribution supported that these populations potentially had expansion after bottleneck effects. These results imply that *T. ciliata* complex could undergo recent expansion in some local regions. One caution is that we only used nrDNA ITS sequences to detect bottleneck effects, which could limit the power of disentangling selection and demography due to their similar footprints in DNA polymorphism. The demographic change causes the entire genome variation while selection brings about regional variations along genomes. Thus, further analysis with multiple genes could help to elucidate these two processes [24].

Previous simulations with the model of maximum entropy (MaxEnt) using 19 climate variables predicted that the most adaptive regions for *T. ciliata* would not substantially shift from 2000 to 2050 year, relative to the adaptive regions in 1950–2000 year [64]. Future climate changes could expand northward in some local regions but shrink in southern regions for most subtropical or tropical species, which is often addressed under climate changes [30,65]. Hu [64] showed that annual and seasonal temperatures are the most key factors to influence the distribution of *T. ciliata*, followed by seasonal precipitation. Based on the MaxEnt model, Zhang et al. [66] predicted that the adaptive region for *T. ciliata* var. *ciliata* would potentially expand as climate changes in the future. However, Zhang et al. [67] predicted that the adaptive region for *T. ciliata* var. *pubescens* would shrink in Yunnan Province. These results suggest that the natural distribution of *T. ciliata* complex would not substantially shift in the future.

The phylogeographic patterns derived from both nrDNA and mtDNA markers provide insight into genetic conservation of this endangered species. In general, we need to consider multiple regions because of the significant IBD effects at the global scale and substantial genetic differentiation among regions. In both western and eastern regions, multiple populations deserve conservation because a certain level of population genetic differentiation occurred for both nuclear and organelle markers within each region. Those populations with higher haplotype diversity deserve protection. However, only a couple of populations could be appropriate for conservation in the central region. This is because population genetic differentiation was small for both nuclear and mitochondrial markers. The haplotype diversity within populations was low as well. Gene flow was extensive within the central region.

Division into three regions is probably supported from the results in a provenance trial of *T. ciliata* complex [8]. This experiment was set up in December of 2013 at Yuejin North Experiment Station, South China Agricultural University. The climate condition of the experimental station belongs to the subtropical monsoon marine, with the annual average temperature of 20~22 °C and the annual rainfall of about 1696.5 mm. Eighteen populations investigated in this study were covered, including four populations from the western region (BS, YR, SM, and PW), seven populations from the central region (YF, LL, TL, XL, LD, WM, and XY), and seven populations from the eastern region (HS, NP, LC, XE, CB, GS, and JG). The mean height of one-year-old seedlings was 89.63 ± 3.46 m for the western populations, 83.48 ± 11.32 m for the central populations, and 29.66 ± 10.51 m for the eastern populations. The same pattern occurred for mean diameter at breast heights (DBH), with the largest DBH from the western populations (25.50 ± 0.67 mm), followed by the central populations (24.34 ± 1.87 mm) and then the eastern populations (10.50 ± 2.41 mm). These results imply the appropriateness of classification of three regions. Thus, a comprehensive strategy could be developed by combing patterns of population structure with the information from provenance trials [68,69].

Our results indicate that pollen flow was more frequent than seed flow in the western and central regions but was slightly greater than seed flow in the eastern region. This could be related to the pattern of mating systems of *T. ciliata* complex, which was obtained in a separate study with different samples even in the same location [15]. A complete outcrossing system occurred in the populations from the western and central regions (BS, YR, SM and TL), but a predominantly outcrossing system, with selfing and inbreeding, occurred in the populations from the eastern region (NP and GS) [15]. Partial selfing and inbreeding bring about low-quality of seeds and cause low natural regeneration [7]. As discussed by Zhou et al. [15], practices of conventional forest management, such as selective logging and clear-cutting, could yield interbreeding among relatives. These practices of conventional forest management erode genetic diversity and destroy habitats. Thus, attention to the role of mating system is important for in situ conservation, especially in the eastern region, as implied by this study.

## 5. Conclusions

*T. ciliata* is an endangered tree species in China due to over cutting and its low natural regeneration. Currently, an important concern is its genetic conservation. In this study, we used both mtDNA and nrDNA ITS markers to elucidate the phylogeographic pattern of *T. ciliata* complex. We investigated twenty-nine populations that covered the range-wide distribution of *T. ciliata* complex in China. Specific conclusions could be drawn below: (1) Haplotypes of mtDNA markers were not randomly distributed in space and phylogeographic structure existed. (2) Population genetic differentiation was significant and most genetic variation occurred among populations. Isolations by distance (IBD) and by elevation (IBE) were present at the global scale. (3) Phylogenetic relationship derived from mtDNA markers indicated three genetically distinct regions each without significant IBD effects. The results from nrDNA ITS sequences showed two regions, consolidating previous findings using nrDNA SRAP and SSR markers. (4) The haplotype diversity was the largest in the western region, followed by the eastern region and the central region. Nucleotide diversity was the smallest in the central region. (5) The rate of pollen to seed flow was large in the western region but small in the central region. The rate of pollen to seed flow was slightly greater than 1 in the eastern region. (6) Most populations did not undergo expansion, with only a few populations showing expansion after bottleneck effects. Based on these phylogeographic patterns, we discussed a strategy of region-based genetic conservation and proposed to conserve multiple populations in the western and eastern regions and a few populations in the central region.

## Figures and Tables

**Table 1:** Locations and sample sizes of 29 populations of *T. ciliata* complex.

Location	Code	Sample Size	Longitude (E)	Latitude (N)	Elevation (m)
Huangshan, Anhui	HS	6	118.08	30.16	499
Jingxian, Anhui	JX	15	118.24	30.41	366
Nanping, Fujian	NP	10	118.10	26.38	800
Lechang, Guangdong	LC	15	113.20	25.07	359
Yunfu, Guangdong	YF	18	111.34	22.46	340
Longlin, Guangxi	LL	10	105.20	24.46	624
Tianlin, Guangxi	TL	26	106.13	24.17	792
Xilin, Guangxi	XL	27	105.05	24.29	899
Luodian, Guizhou	LD	14	106.44	25.25	814
Ceheng, Guizhou	CH	22	105.48	24.59	730
Wangmo, Guizhou	WM	30	106.05	25.10	500
Xingyi, Guizhou	XY	15	104.54	25.06	1160
Xuanen, Hubei	XE	9	109.28	30.17	632
Chengbu, Hunan	CB	25	111.28	27.14	737
Hupingshan, Hunan	HP	3	111.41	29.01	436
Xinning, Hunan	XN	30	109.44	28.18	650
Maotoushan, Jiangxi	MT	14	117.03	27.41	1000
Wuyishan, Fujian	WY	9	117.42	28.18	1200
Guanshan, Jiangxi	GS	14	115.26	27.19	337
Jinggangshan, Jiangxi	JG	17	114.17	26.44	400
Jiulianshan, Jiangxi	JL	18	114.47	24.54	610
Dechang, Sichuan	DC	29	102.32	26.40	1325
Huidong, Sichuan	HD	30	102.09	27.23	1862
Baoshan, Yunnan	BS	11	99.06	25.04	1513
Yongren, Yunnan	YR	29	101.32	25.01	1539
Simao, Yunnan	SM	16	100.58	22.46	1317
Puwen, Yunnan	PW	19	101.04	22.23	890
Xianju, Zhejiang	XJ	7	119.92	28.45	600
Suichang, Zhejiang	SC	12	119.25	28.59	510

**Table 2:** Genetic diversity of *T. ciliata* complex derived from the concatenated sequences of mitochondrial *cox1−nad*1 and *26S−rRNA−tRNA−*Leu, and the nrDNA ITS sequences.

Population	mtDNA Marker		Haploid nrDNA ITS		Tajima’s D (*p*-Value)	Fu’s F (*p*-Value)
	*h*	*π*	*π*	*θ*		
HS	0.500	0.00108	0.02700	0.036	−0.73 (0.29)	2.07 (0.76)
JX	0.124	0.00032	0.00888	0.018	−1.79 (0.03)	−0.75 (0.35)
NP	0.500	0.00045	0.00413	0.006	−0.76 (0.24)	−1.54 (0.14)
LC	0.240	0.00065	0.01512	0.019	−0.88 (0.18)	−1.11 (0.27)
YF	0.401	0.00171	0.00906	0.014	−1.50 (0.04)	−1.00 (0.31)
LL	0.000	0.0000	0.00528	0.008	−0.35 (0.41)	−0.88 (0.28)
TL	0.074	0.00019	0.00609	0.009	−0.93 (0.21)	−16.71 (0.00)
XL	0.000	0.0000	0.00542	0.013	−1.90 (0.02)	−9.32 (0.00)
LD	0.000	0.0000	0.00481	0.008	−1.58 (0.04)	−4.70 (0.00)
CH	0.000	0.0000	0.00691	0.011	−0.98 (0.19)	−9.27 (0.00)
WM	0.180	0.00015	0.00614	0.014	−1.88 (0.01)	−10.88 (0.00)
XY	0.000	0.0000	0.01136	0.019	−1.36 (0.08)	−8.81 (0.00)
XE	0.346	0.00031	0.00186	0.003	−0.80 (0.24)	−0.40 (0.38)
CB	0.403	0.00034	0.00617	0.010	−1.15 (0.14)	−2.22 (0.17)
HP	0.000	0.0000	0.00220	0.003	NA	NA
XN	0.000	0.0000	0.00266	0.005	−1.16 (0.12)	1.31 (0.79)
MT	0.000	0.0000	0.00246	0.003	−0.20 (0.62)	1.31 (0.77)
WY	0.370	0.00148	0.02526	0.032	−0.65 (0.29)	1.62 (0.73)
GS	0.459	0.00108	0.00360	0.006	−1.24 (0.10)	−1.41 (0.18)
JG	0.381	0.00173	0.01811	0.026	−1.11 (0.11)	−0.09 (0.46)
JL	0.444	0.00038	0.00441	0.012	−1.74 (0.03)	−1.73 (0.18)
DC	0.186	0.00015	0.00786	0.014	−1.27 (0.10)	−6.69 (0.01)
HD	0.000	0.0000	0.00515	0.008	−0.92 (0.20)	−8.37 (0.00)
BS	0.000	0.0000	0.03222	0.038	−0.72 (0.22)	2.12 (0.83)
YR	0.000	0.0000	0.00590	0.009	−0.75 (0.26)	−7.17 (0.00)
SM	0.508	0.00077	0.00515	0.008	−1.52 (0.06)	−6.90 (0.00)
PW	0.388	0.00033	0.00527	0.007	−0.75 (0.25)	−0.59 (0.39)
XJ	0.000	0.0000	0.00110	0.002	−1.61 (0.04)	−0.13 (0.44)
SC	0.000	0.0000	0.00330	0.005	−0.18 (0.46)	−0.73 (0.34)

**Table 3:** Analysis of molecular variance (AMOVA) using mtDNA and nrDNA ITS sequences of *T. ciliata* complex.

Marker	Source of Variation	d.f.	Sum of Square	Variance Component	Percentage of Variance (%)	*Φ_st_*	*p*-Value
*cox1−nad*1	Among populations	28	1599.283	3.3129	88.58	0.8858	0.00
	Within populations	471	201.153	0.4271	11.42		
	Total	499	1800.436	3.7400			
*26S−rRNA−tRNA−*Leu	Among populations	28	2527.316	5.2370	89.01	0.8901	0.00
	Within populations	471	304.612	0.6467	10.99		
	Total	499	2831.928	5.8837			
*cox1−nad*1 and *26S−rRNA−tRNA−*Leu	Among populations	28	4126.600	8.5499	88.84	0.8884	0.00
	Within populations	471	505.764	1.0738	11.16		
	Total	499	4632.364	9.6237			
ITS	Among populations	28	3192.408	6.9508	71.43	0.7143	0.00
	Within populations	438	1217.771	2.7803	28.57		
	Total	466	4410.180	9.7311			

**Table 4:** Estimates of the relative rate of pollen to seed flow in local and global regions of *T. ciliata* complex.

	Western Region	Central Region	Western and Central Region	Eastern Region	Global Region
*F_st_*_(*n*)_	0.1362	0.0506	0.1068	0.1810	0.6994
*Sd*(*F_st_*_(*n*)_)	0.0016	0.0038	0.0003	0.0247	0.0079
*F_st_*_(*m*)_	0.8405	0.0262	0.8566	0.5360	0.8870
*Sd*(*F_st_*_(*m*)_)	0.0600	0.0011	0.0022	0.1267	0.0077
*m_P_/m_S_*	31.4134	-1.4950	47.9628	3.2270	1.3741
*Sd*(*m_P_/m_S_*)	13.2330	0.0454	0.8091	2.5527	0.2925

## Data Availability

All data sets used in this study were provided in Tables S2 and S3 in Supplementary Materials.

## Supplementary Materials

The following supporting information can be downloaded at: https://www.mdpi.com/article/10.3390/genes14010116/s1. Table S1: Twenty pairs of primers from mitochondrial DNA tested in *T. ciliata* complex. Table S2: Five hundred samples of alignment sequences each of which was a concatenated sequence of *cox*1−*nad*1 and *26S*−*rRNA*−*tRNA*−Leu segments. Table S3: Four hundred and sixty-seven samples of ITS alignment sequences. Table S4: Estimates of parameters of mismatch distribution and statistical tests.
