# Supplementary material for: Phylogeography of Toona ciliata (Meliaceae) Complex in China Inferred from Cytonuclear Markers
Source: Genes (Basel). 2022 Dec 31;14(1):116. doi: 10.3390/genes14010116 (PMC9858616; doi:10.3390/genes14010116)
Supplement: Supplementary file 1 [file genes-14-00116-s001.zip › Supplementary_Materials_20221129.pdf]

Phylogeography of *Toona ciliata* (Meliaceae) complex in China inferred from cytonuclear markers

Yu Xiao[1,2], Xin-Xin Zhang [1,2], Ying Hu [1,2], Xi Wang [1,2], Pei Li[1,2], Zi-Han He [1,2], Yan-Wen Lv[1,2], Xiao-Yang Chen [1,2] and Xin-Sheng Hu[1,2*]

1 College of Forestry and Landscape Architecture, South China Agricultural University, Guangzhou 510642, China

2 Guangdong Key Laboratory for Innovative Development and Utilization of Forest Plant Germplasm, Guangzhou 510642, China

* Correspondence: Xin-Sheng Hu; Email: xinsheng@scau.edu.cn

Running title: Phylogeography of *Toona ciliata*

Table S1. Twenty pairs of primers from mitochondrial DNA tested in *Toona ciliata* complex

Primer pair	Forward and reverse primer sequences	Annealing temperature	Reference
*atp*9F-*atp*9R	CCAAGTGAGATGTCCAAGAT<br>CTTCGGTTAGAGCAAAGCC	50	Duminil *et al.*, 2002
*ccb*203F-*ccb*203R	ASGTTCTACGGACCGATGCC<br>CACGGGGAGGGAGCRGGCGA	56	Duminil *et al.*, 2002
*ccb*206F-*ccb*206R	TCAATCTTGTRAACTAATCG<br>CYYCTCCACACCAATCACGA	62	Duminil *et al.*, 2002
*ccb*256F-*ccb*256R	GGAAGTTAGCAAAGTTAGAC<br>TTGTTCTTAACAGCGATGGC	56	Duminil *et al.*, 2002
*cox*2/1-*cox*2/2	TTTTCTTCCTCATTCTKATTT<br>CCACTCTATTGTCCACTTCTA	50	Dumolin-Lapegue *et al.*, 1997
*cox*3F-*cox*3R	CCGTAGGAGGTGTGATGT<br>CTCCCCACCAATAGATAGAG	58	Duminil *et al.*, 2002
*orf*25F-*orf*25R	AAGACCRCCAAGCYYTCTCG<br>TTGCTGCTATTCTATCTATT	50	Duminil *et al.*, 2002
*rpl*5F-*rpl*5R	AGTGGTAAAGTCTCATCT<br>ATYGTGTGAAATAAGAGTAG	50	Duminil *et al.*, 2002
*rps*4F-*rps*4R	CSTTTCYGCTCCGAAGAG<br>TCTCCGAAGATTGAGG	58	Duminil *et al.*, 2002
*rps*12-1-*nad*3-2	TTTCTTCTCTACCATGACGA<br>TGATCCYACTCGGTSTTCCT	50	Duminil *et al.*, 2002
*rrn*5-*rrn*18-1	GAGGTCGGAATGGGATCGGG<br>GGGTGAAGTCGTAACAAGGT	58	Duminil *et al.*, 2002
*mh*44_F-*mh*44_R	ATGACTGGAAGAATTGCTCAC<br>TTCACTTGATACTCACCCCC	55	Duminil *et al.*, 2002
*nad*1/B-*nad*1/C	GCATTACGATCTGCAGCTCA<br>GGAGCTCGATTAGTTTCTGC	57.5	Demesure *et al.*, 1995
*nad*4/1-*nad*4/2	CAGTGGGTTGGTCTGGTATG<br>TCATATGGGCTACTGAGGAG	57.5	Demesure *et al.*, 1995
*hc_mt*1F-*hc_mt*1R	AGGGTGGCTATTTCTCGCTC<br>CGAGAAAGAATAGAATCAAGGG	52	This study
*hc_mt*2F-*hc_mt*2R	TGCTACTGCTGCTACCATGA<br>GCACCAGCAAAGCAAAGCT	50	This study
*hc*_F3-*hc*_R3	AACATAGAACTGAGGTAATG<br>ATTACGCCAAAACTGATGCT	50	This study
26SrRNA-tRNA-Leu	AACATAGAACTGAGGTAATG<br>ATTACGCCAAAACTGATGCT	50	This study
26SrRNA-tRNA-Leu	ACGGATACTTTGTCATTAGA<br>TTTATGTGGTTCCGACTGTA	50	This study
cox1-nad1	ACAAACAGTTAGTAAGGTCA<br>TTTCCAGTCTTCAAATCGGT	50	This study

Table S4. Estimates of parameters of mismatch distribution and statistical tests #

Population	θ_0	θ_1	$\tau(t)$	SSD (P-value)	Rag (P-value)
JX	7.794	16.549	1.977	0.059 (0.20)	0.121 (0.11)
YF	1.995	23.125	2.125	0.014 (0.44)	0.042 (0.48)
XL	1.552	37.285	3.131	0.002 (0.84)	0.013 (0.86)
LD	0.028	14.214	4.205	0.003 (0.94)	0.020 (0.92)
CH	0.171	122.344	5.359	0.004 (0.51)	0.020 (0.51)
WM	1.872	50.859	2.607	0.005 (0.53)	0.023 (0.55)
XY	-	-	-	-	-
JL	0.021	12.109	3.418	0.043 (0.07)	0.136 (0.04)
DC	2.164	41.680	3.867	0.003 (0.69)	0.010 (0.87)
HD	0.002	15.073	4.576	0.007 (0.61)	0.023 (0.76)
YR	0.011	15.264	4.773	0.001 (0.95)	0.010 (0.99)
SM	0.068	42.802	3.582	0.002 (0.90)	0.022 (0.84)
XJ	-	-	-	-	-
TL	-	-	-	-	-

#: SSD: the sum of squared deviation (SSD); Rag: Harpending's raggedness index; θ_0=$2N_0\mu$, θ_1=$2N_1\mu$, where N_0 and N_1 are the population sizes before and after population expansion; τ (t): the time elapsed since a sudden expansion. "-": appropriate estimates were not derived from sequence data. Populations XJ and TL: Estimates were not available.

Table S2: Five hundred samples of alignment sequences each of which was a concatenated sequence of *cox*1-*nad*1 and *26S-rRNA*-*tRNA*-Leu segments

Table S3: Four hundred and sixty-seven samples of ITS alignment sequences
